# Vaccination as a Preventive of Small-pox

**Published:** 1851-06

**Authors:** 


*Vaccination as a Preventive of Small-pox.*—Dr. Alexander Knox concludes a highly interesting paper on the existing state of our knowledge of vaccination and revaccination as preventive of small-pox (*Lond. Journ. Med.*, Dec. 1850), with the following practical deductions, suggested by a careful reconsideration of the subject, and which are well calculated to reassure confidence in the protective influence of cow-pox.

"1. It appears to have been satisfactorily demonstrated, that secondary vaccinations have succeeded in a considerable proportion of the cases in which they have been resorted to.

2. It also appears that small-pox has prevailed of late years to an increased extent.

3. The results in question have been attributed, partly to a dimution of energy in the vaccine infection, caused by repeated transmission through the human subject, and partly to the alleged tendency of the immunity conferred by cow-pock, to wear out of the system, after an uncertain period from the date of vaccination.

4. Both the success of revaccination, and the increased prevalence of casual small-pox, appear, however, to have been exaggerated in the popular belief; and, at any rate, the facts seem explicable, in a great measure, without resorting to the hypothesis just stated, by attributing them in part to the imperfect performance, or the entire neglect of vaccination; in part to the temporary tendency to increased diffusion, at distant and uncertain periods of time, which characterizes all epidemic diseases; and, finally, to peculiarities of constitution, which render many iudividuals absolutely insusceptible of being protected against a secondary attack, either by vaccination or by inoculated or natural small-pox.

5. It has been proposed to re-introduce variolous inoculation as a certain remedy for the occasional failure of vaccination; but the superior efficacy of the practice is not only questionable, but its indiscriminate employment has been proved to be dangerous, and destructive of human life, and is therefore highly to be deprecated.

6. Revaccination, however, may be prudently recommended, not only as innocuous in itself, but also, on various grounds, as positively advantageous, even by those who question the gradual extinction of the protective influence of cow-pock.

7. It does not appear that genuine vaccination has lost any of the efficacy which at any time really appertained to it; and it still remains to be demonstrated that it is not capable of conferring, to the end of life, complete immunity from the horrors of small-pox, on a large majority of all the individuals fully submitted to its influence.

8. Even where vaccination fails to prevent a secondary attack, the consecutive disease, in general, assumes a mild and modified form, although, in some instances, it may be sufficiently severe to leave the countenance marked with scars, and still more rarely to terminate in death: but fatal cases from secondary small-pox do not seem to be more frequent after vaccination than after a primary attack of the natural disease.

9. On the whole, it is respectfully maintained that cow-pock, imparted in the most efficient manner of which it is capable, by vaccination, and, under certain circumstances, by revaccination, is the most eligible safeguard within our power against small-pox; and that it will prove effectual in most constitutions, not inherently insusceptible of protection, by any means whatever."—*Ibid.*

